# 

**Published:** 1879-03

**Authors:** 


CONTENTS.

| | Page |
|---|---|
| Dirt | 751 |
| Physiology | 752 |
| Letter of John Randolph | 752 |
| Bible Confirmations | 755 |
| Anecdote of Thomas Paine | 756 |
| Be Courteous | 757 |
| House Building | 759 |
| Farmers' Wives Overtaxed | 760 |
| Never | 768 |
| Eyes and Cold Water | 768 |
| What shall we Eat and Drink | 769 |
| Don't Get Discouraged | 771 |
| Ozone | 772 |
| The Longest Lives | 774 |
| Mind and Health | 774 |
| Old Age | 775 |
| How People Take Cold | 775 |
| Hydrophobia | 776 |

